# The Transcription Factor ZNF683/HOBIT Regulates Human NK-Cell Development

**DOI:** 10.3389/fimmu.2017.00535

**Published:** 2017-05-15

**Authors:** Mirte Post, Angelica Cuapio, Markus Osl, Dorit Lehmann, Ulrike Resch, David M. Davies, Martin Bilban, Bernhard Schlechta, Wolfgang Eppel, Amit Nathwani, Dagmar Stoiber, Jan Spanholtz, Emilio Casanova, Erhard Hofer

**Affiliations:** ^1^Institute of Vascular Biology and Thrombosis Research, Center of Physiology and Pharmacology, Medical University of Vienna, Vienna, Austria; ^2^Department of Oncology, University College London Cancer Institute, London, UK; ^3^Department of Laboratory Medicine, Medical University of Vienna, Vienna, Austria; ^4^Department of Obstetrics and Gynecology, Medical University of Vienna, Vienna, Austria; ^5^Ludwig Boltzmann Institute of Cancer Research, Vienna, Austria; ^6^Institute of Pharmacology, Center of Physiology and Pharmacology, Medical University of Vienna, Vienna, Austria; ^7^Glycostem Therapeutics, Oss, Netherlands; ^8^Institute of Physiology, Center of Physiology and Pharmacology, Comprehensive Cancer Center, Medical University of Vienna, Vienna, Austria

**Keywords:** ZNF683/HOBIT, natural killer cells, CD56, *ex vivo* differentiation, NK-cell development

## Abstract

We identified *ZNF683/HOBIT* as the most highly upregulated transcription factor gene during *ex vivo* differentiation of human CD34^+^ cord blood progenitor cells to CD56^+^ natural killer (NK) cells. ZNF683/HOBIT mRNA was preferentially expressed in NK cells compared to other human peripheral blood lymphocytes and monocytes. During *ex vivo* differentiation, ZNF683/HOBIT mRNA started to increase shortly after addition of IL-15 and further accumulated in parallel to the generation of CD56^+^ NK cells. shRNA-mediated knockdown of ZNF683/HOBIT resulted in a substantial reduction of CD56^−^CD14^−^ NK-cell progenitors and the following generation of CD56^+^ NK cells was largely abrogated. The few CD56^+^ NK cells, which escaped the developmental inhibition in the ZNF683/HOBIT knockdown cultures, displayed normal levels of NKG2A and KIR receptors. Functional analyses of these cells showed no differences in degranulation capacity from control cultures. However, the proportion of IFN-γ-producing cells appeared to be increased upon ZNF683/HOBIT knockdown. These results indicate a key role of ZNF683/HOBIT for the differentiation of the human NK-cell lineage and further suggest a potential negative control on IFN-γ production in more mature human NK cells.

## Introduction

Natural killer (NK) cells are the third largest group of lymphocytes in peripheral blood and an important component of the first line of immune defense. They act against a wide spectrum of virally infected and neoplastic cells by direct killing of these cells or production of cytokines, such as IFN-γ. As components of the innate part of the immune system, they display immediate reactivity and do not require prior sensitization (1, 2). This traditional characterization of NK cells has been expanded over the recent years as they have been described to be able to incorporate features previously thought to be restricted to the adaptive immune system, such as interaction with dendritic cells and immunological memory (3–5). In contrast to the adaptive T and B lymphocytes, NK cells lack somatically recombined and clonally distributed antigen receptors, and their activity is controlled by a varied repertoire of germline-encoded inhibitory and activating receptors (6). Recently, additional tissue-resident subsets of innate lymphoid cells (ILCs), distinct from NK cells, became apparent and significantly expanded the complexity of innate lymphoid lineages. Whereas conventional NK cells resemble cytotoxic T lymphocytes in many aspects, ILCs 1–3 rather mirror T helper-like cells (7).

Based on currently available data, the relationship between the different innate and adaptive lymphoid lineages is reflected by their initial common differentiation from the hematopoietic stem cell (HSC) and in similarities of their transcriptional networks. According to the current hypothesis, the HSC develops through a multipotent progenitor to a common lymphoid progenitor (CLP) (8). This CLP can further differentiate into adaptive lymphocytes under the control of E-proteins, whereas the development of innate lymphocytes requires antagonism of E-proteins and likely proceeds through a common innate lymphoid progenitor (9, 10). The following pre-NK progenitor (NKP) stage restricts the differentiating cells to the NK lineage and develops into NKP and subsequently into immature NK (iNK) cells. The final step involves maturation from the iNK cells to mature NK (mNK) cells, both stages expressing the NK marker CD56 (11, 12).

Substantial data have been obtained to identify key transcription factors essential for the differentiation of adaptive and innate lymphocytes. A common theme appears to be the mutual inhibition of factors determining different lineages. For example, EBF strongly inhibits ID2 expression, thereby allowing E2A to function during B-cell development. In addition, EBF and PAX5 support B-cell differentiation by repressing additional critical regulators of T-cell and ILC differentiation, such as NOTCH1, GATA-3, and TCF-1 (8, 9). Conversely, all ILCs including NK cells are dependent for their differentiation on ID2 that heterodimerizes with E proteins and neutralizes their activity (10). Subsequently, a complex network of transcription factors guides the cells through the distinct steps of NK-cell differentiation (13). The most important transcription factors for the early stages of murine NK-cell development include STAT5, two ETS family members (PU.1 and ETS-1), and NFIL3 (also known as E4BP4) (14–17). The maturation stage from iNK to mNK cells and NK cells’ function are coordinated by BLIMP-1, T-BET, EOMES, and MEF among others (18–20). Compared to the data obtained from the murine system, experimental evidence on transcription factors governing human NK-cell development is far less available. This has been partially caused by the lack of an easy and robust system to mimic human NK-cell differentiation from HSC *ex vivo*. Of the few transcription factors described so far, TOX1 and 2 are important in the early stages, T-BET and BLIMP-1 both play critical roles in the later phases and effector functions of human NK cells (21–24). From the currently available data, it appears that the precise function and sequential order of transcription factors directing NK-cell development may to some extent differ between mice and humans.

Due to their important role in immunosurveillance, NK cells and their modulation are currently being explored as a therapeutic approach in a wide variety of cancers, autoimmune diseases, allergies, and transplantation (1, 25). These attempts have led to the development of methods for the specific modulation of endogenous NK cells as well as for *ex vivo* amplification of NK cells from patients or allogeneic donors for NK cell-infusion therapies. The obtained results show clear benefits of NK cell-based therapies, in comparison to T lymphocyte-based, including a good tolerance of allogeneic NK cells by the patients and the lack of a graft-versus-host disease (1, 25). Different strategies are exploited to obtain a sufficient number of NK cells for infusion therapies, including cytokine- and/or feeder cell-mediated expansion of peripheral blood NK cells as well as *ex vivo* differentiation from cord or peripheral blood-derived HSC (26, 27). In this regard, we have previously analyzed a feeder cell-free *ex vivo* system to generate large-scale therapeutic NK cells from cord blood stem cells that faithfully reproduces different steps of human NK-cell differentiation (28). This system is, therefore, ideally suited to investigate human NK-cell differentiation in addition to being a reliable method to generate NK cells for therapy (29, 30).

Here, we used this *ex vivo* differentiation system for a complete transcriptomic profiling of cells in different stages of NK-cell development. We identified the 20 most differentially expressed transcription factor genes and confirmed ZNF683/HOBIT mRNA as the highest upregulated transcription factor mRNA. The analysis of different human peripheral blood cell types showed preferential expression of ZNF683/HOBIT mRNA in NK cells. Then, we analyzed the role of ZNF683/HOBIT during NK-cell differentiation in more detail. In the *ex vivo* differentiation system, shRNA-mediated knockdown of ZNF683/HOBIT significantly reduced CD56^−^CD14^−^ NKPs up to day 21 of culture and nearly abrogated the following generation of CD56^+^ NK cells. The few CD56^+^ cells that continued to mature displayed normal levels of NKG2A and KIR as well as degranulation capacities similar to control cells. However, the number of IFN-γ-producing cells significantly increased upon ZNF683/HOBIT knockdown. Taken together, these results support that ZNF683/HOBIT is a key regulator of early stages of human NK-cell differentiation and, in later stages, may function to repress IFN-γ production.

## Materials and Methods

### *Ex Vivo* Differentiation of CD34^+^ Stem Cells into NK Cells

Human umbilical cord blood samples were obtained at birth after full-term delivery from the Department of Obstetrics and Gynecology of the University Hospital of Vienna, Austria. Cord blood mononuclear cells (CBMCs) were collected by Ficoll density gradient centrifugation (Lympholyte Cell Separation Media, human, Cedarlane^®^, Burlington, ON, Canada). Stem cells were isolated from CBMCs according to manufacturer’s protocol, using a magnetic bead-based CD34^+^ isolation kit (CD34 MicroBead Kit, human; Miltenyi Biotec, Bergisch Gladbach, Germany). The purity of the stem cells was evaluated by flow cytometry (CD34^+^ reached at least 95% purity) after which the cells were cultured as previously described (28, 30). In short, stem cells at an initial density of 10^5^ cells/ml were seeded into 6-well plates (Corning Incorporated, Corning, NY, USA) for 10 days in basal expansion medium (GBGM©; Glycostem Therapeutics, Oss, The Netherlands) supplemented with stem cell factor (SCF), interleukin-7 (IL-7), thrombopoietin (TPO), and FMS-like tyrosine kinase 3 ligand (Flt3L); all factors at a concentration of 25 ng/ml (CellGro^®^, CellGenix GmbH, Freiburg, Germany) and granulocyte colony-stimulating factor (G-CSF, 250 pg/ml; Stemcell Technologies, Vancouver, BC, Canada), granulocyte-macrophage colony-stimulating factor (GM-CSF, 10 pg/ml; Stemcell Technologies), and IL-6 (50 pg/ml; CellGenix GmbH). At day 10, TPO was replaced with IL-15 (20 ng/ml; CellGro^®^, CellGenix GmbH), and at day 14, Flt3L with IL-2 (1,000 U/mL; Chiron Corporation, Emeryville, CA, USA) by refreshing half of the medium. After a total culture period of 35 days, a regular culture consisted of >95% CD56^+^CD3^−^ NK cells as evaluated by flow cytometry.

### Microarray

Total RNA was extracted from cells at different time points of the *ex vivo* culture and transcribed into cDNA using the GeneChip^®^ Whole Transcript Sense Target Labeling Kit (Affymetrix, High Wycombe, UK). The labeled cDNA was hybridized to GeneChip Human Gene 1.0ST Arrays, and the arrays were scanned and analyzed according to protocols of the manufacturer^1^ as described in Ref. (31). Robust multiarray average signal extraction and normalization were performed, as detailed at the Bioconductor website^2^ (32). The microarray data have been submitted to the GEO database under the accession number GSE95018.

### RNA Sampling, cDNA Synthesis, and Real-time RT-PCR

Cultured cells (5 × 10^5^) were lysed in Trizol (QIAzol Lysis Reagent, Qiagen Biosciences, MD, USA) and stored at −80°C. Total RNA was extracted following ThermoFishers manual^3^, and 1 µg RNA was used for cDNA synthesis according to manufacturer’s protocol (RevertAid H Minus First Strand cDNA Synthesis Kit, ThermoFisher Scientific, MA, USA). Two hundred nanograms of transcribed cDNA were analyzed by real-time PCR using the KAPA SYBR FAST UNIVERSAL kit (Kapa Biosystems, Inc., Wilmington, MA, USA) and primer sequences as shown in Table S3 in Supplementary Material. As internal controls, primers for either hypoxanthine-guanine phosphoribosyltransferase or β-actin were used. Samples were measured and analyzed with QIAGEN’s real-time PCR cycler and corresponding software (Rotor-Gene Q, Qiagen, Hilden, Germany).

### Western Blot

For protein-expression analysis, cell pellets (5 × 10^5^ cells/sample) were resuspended in 2× Laemmli buffer, boiled for 10 min at 95°C and subsequently separated by SDS-polyacrylamide gel electrophoresis (10% Bis-Tris gels, Acrylamide:Bis 37.5:1) and transferred to a nitrocellulose membrane (Amersham Protran Supported 0.45 µm NC, GE Healthcare Europe GmbH, Eindhoven, The Netherlands) by semi-dry electrophoretic blotting in Towbin buffer with 20% methanol (PerfectBlue, “Semi-Dry” Electroblotter Sedec, Peqlab, Southampton, UK). The membrane was blocked with 5% non-fat dry milk in TBS-T (0.2% Tween in TBS) followed by overnight incubation at 4°C with primary anti-ZNF683 antibodies (goat-anti-ZNF683 antibody C-12, 1:500, Santa Cruz Biotechnology, Heidelberg, Germany) or (mouse-anti-ZNF683 antibody, 1:500, Sigma-Aldrich, Saint Louis, MO, USA) and as control anti-GAPDH (mouse-anti-GAPDH, 1:10,000, Merck KGaA, Darmstadt, Germany). This was followed by 2 h incubation at room temperature (RT) with secondary antibodies, all 1:5,000 (donkey-anti-goat, Santa Cruz Biotechnology or goat-anti-mouse, Thermo Fisher Scientific) and corresponding washing steps with TBS-T.

### Lentiviral Vectors

To generate a lentiviral construct for knockdown studies, a sense oligo, 5′-TGGAAACACATGGGCTATGACATTTCAAGAGAATGTCATAGCCCATGTGTTTCTTTTTTC-3′, corresponding with the sequence “GAAACACATGGGCTATGACAT” to position 1,402 to 1,412 from the start ATG of *ZNF683/HOBIT* cDNA (NCBI Reference Sequence: NM_001114759.2) and a corresponding antisense oligo, 5′-TCGAGAAAAAAGAAACACATGGGCTATGACATTCTCTTGAAATGTCATAGCCCATGTGTTTCCA-3′ were synthesized (Integrated DNA Technologies Inc., Coralville, IA, USA) as complementary overlapping oligos, with a *Xho1* overhang at the 5′end of the antisense oligo, as detailed.^4^ The complementary oligos were annealed and subsequently ligated into the LeGO-G/BSD lentiviral vector (LeGO-G/BSD was a gift from Boris Fehse, Addgene plasmid #27354) that had been digested with *Hpa*I and *Xho*I (New England Biolabs, Ipswich, MA, USA). The ligated product was transformed into the *E. coli* strain Stbl3 (New England Biolabs, Ipswich, MA, USA), recombinant colonies were selected, and plasmid-DNA isolated and verified by restriction digestion and Sanger sequencing. As a control, oligos containing a scrambled shRNA were synthesized and cloned into the LeGO-G/BSD vector using an identical strategy. The control shRNA sequence was obtained from the “Open Biosystems pGIPZ shRNAmir library” situated at University College London.^5^ To confirm the functioning of the shRNA, shRNA expression plasmids were co-transfected into HEK293 cells in the absence or presence of a commercially available ZNF683/HOBIT expression plasmid (ORF of ZNF683, transcript variant 1, in pEnter, with C-terminal Flag and His tag; Vigene Biosciences, Rockville, MD, USA) employing the CaPO_4_ method using 4 µ*g* of total DNA/6-well. Six hours after, transfection medium was changed, and cells were harvested 96 h after transfection by either direct lysis in Trizol (Qiagen) for RNA isolation or 1× Laemmli sample buffer for protein-expression analysis.

### Virus Production

Virus production and all experiments involving the generated recombinant viruses were performed in a separate room under biosafety level (BSL)-2 conditions following the instructions given by the corresponding directives of the European Union (Council Directive 90/679/EEC) and the Austrian government (GTG-BGBl. Nr. 510/1994 and 114/2012). The specific work with the replication-defective human lentiviruses was registered at the Austrian Ministry of Science, Research and Economy (BMWFW-5.011/009-WF/V/3b/2015).

To produce lentiviruses, HEK293T cells (ATCC # CRL-11268) were seeded at 10^7^ cells per 15 cm^2^ tissue culture plate in 15 ml DMEM (DMEM/high glucose, GE Healthcare) supplemented with 10% FBS and glutamine (200 mM, Sigma-Aldrich). After 24 h, 5 ml DMEM was added, and the 60–70% dense cultures were transfected with the LeGO-G/BSD-*ZNF683/HOBIT*-shRNA expression plasmid plus three complementing plasmids providing the essential *Gag, Pol, Rev*, and *Tat* gene products missing in the replication-defective virus. To this end, the following 2 transfection solutions were prepared: (1) DMEM with 10% polyethyleneimine (PEI, Polysciences, Warrington, PA, USA) (1.5 ml/15 cm^2^ plate) and (2) DMEM with 2.3 µg pCAG-KGP3R, 1.2 µg pCAG-RTR2, 2.5 µg pCAG-VSVg, and 24 µg vector of interest (in total 30 µg plasmids in 1.55 ml/15 cm^2^ plate). After 15 min incubation at RT, solution 2 was sterile filtered and added to solution 1. The combined solutions were added dropwise to HEK293T cells at a density of 70%. After 12 h, the transfection mix was replaced with fresh medium (12 ml DMEM/15 cm^2^ plate). The supernatants were harvested after 48 and 72 h post-transfection. To remove cell debris, supernatants were centrifuged (1,500 rpm, 5 min, 4°C), filtered (Puradisc FP30mm cellulose acetate syringe filter, 0.45 µm, sterile, GE Healthcare Life Sciences), and finally the virus particles were concentrated by ultracentrifugation for 90 min at 4°C at 25,000 rpm/76,000 × *g* in a SW-32 rotor equipped with 32 ml open-top thickwall polycarbonate tubes in a XL-70 ultracentrifuge (Beckman Coulter, Mystic, CT, USA). After ultracentrifugation, the supernatant was discarded leaving a 100 µl drop at the bottom of the tube. The virus particles were gently resuspended after keeping the tubes on ice for 1 h, aliquoted, and stored at −80°C until use.

### Virus Transduction of CD34^+^ Stem Cells

After culturing freshly isolated cord blood CD34^+^ stem cells for 5 days in basal expansion medium supplemented with SCF, IL-7, TPO, and Flt3L as described under “*Ex vivo differentiation of CD34^+^stem cells into NK cells*,” the expanded cells were transduced with lentiviral particles. To this end, 24-well plates (multi-well plate for suspension culture, Greiner Bio-one GmbH, Frickenhausen, Germany) were coated with 8 µg retronectin/500 μl/well (recombinant human fibronectin fragment, Takara Bio Inc., Shiga, Japan) 24 h prior virus transduction and stored at 4°C until use. The retronectin solution was removed, 250 µl GBGM containing virus particles (40 Transduction Units/cell) were added per well, and the plate was centrifuged at 4,000 rpm/1,900 × *g* for 2 h at 4°C. Immediately after centrifugation, the 5 days cultivated CD34^+^ stem cells were added (5 × 10^4^ cells in 250 µl basal expansion medium/well). The following day, 500 µl of basal expansion medium was added. Two days after transduction the cells were centrifuged (1,500 rpm for 5 min), suspended in fresh basal medium, and transferred to new uncoated 24-well cell culture plates (Corning^®^ Costar^®^, Sigma-Aldrich). Transduction efficiency, determined as GFP^+^ cells, was measured by flow cytometry 3 days after transduction, and cultures were continued as described in the first section (for *ex vivo* differentiation). Further cell expansion was calculated as follows. Cell numbers were obtained at the various differentiation stages and divided by the cell numbers for the corresponding GFP^+^ and GFP^−^ fractions measured 3 days after transduction, to correct for different transduction efficiencies.

### Flow Cytometry

Cell surface expression of NK and monocytic markers was monitored using anti-CD56-PeCy7, anti-CD14-PerCPCy5.5 (all BD Biosciences, San Jose, CA, USA), and anti-KIR-PE antibodies (R&D Systems, Vienna, Austria) on a FACS Canto II (BD Biosciences) and data were analyzed using both FACS DIVA software v6.0 (BD Biosciences) and Flowjo v10.0.8 (Tree Star, Yorba Linda, CA, USA). Details on antibodies are given in Table S4 in Supplementary Material.

### Cytotoxicity and IFN-γ Assay

Target K562 and effector NK cells were cocultured at a 1:1 ratio (15 × 10^4^ cells of each cell type) in 200 µl RPMI medium (RPMI 1640 medium, Life Technologies, Carlsbad, CA, USA) in an U-bottom 96-well plate (Greiner Cellstar^®^ 96-well plates, Sigma-Aldrich) in the presence of anti-CD107a-APC (BD Biosciences). Brefeldin A and Monensin (BD Golgiplug and Golgistop, BD Biosciences) were added after 1 h of culture. After an additional 5 h of culture, cells were collected, stained for surface CD56 (anti-CD56-PeCy7, BD Biosciences), subsequently prepared for intracellular staining with IFN-γ (anti-IFN-γ-PE, BD Biosciences) using a fixation/permeabilization solution kit (BD Biosciences), and finally measured on a FACS Canto II.

### Statistical Analysis

Statistical analysis was performed with Prism 6 software (GraphPad, San Diego, CA, USA) using Student’s *t*-test or a two-way ANOVA as indicated in Figure Legends. A *p*-value of 0.05 was considered as statistically significant.

## Results

### *ZNF683/HOBIT* Is the Most Highly Upregulated Transcription Factor Gene During *Ex Vivo* Differentiation of Human NK Cells

**Figure 1:**
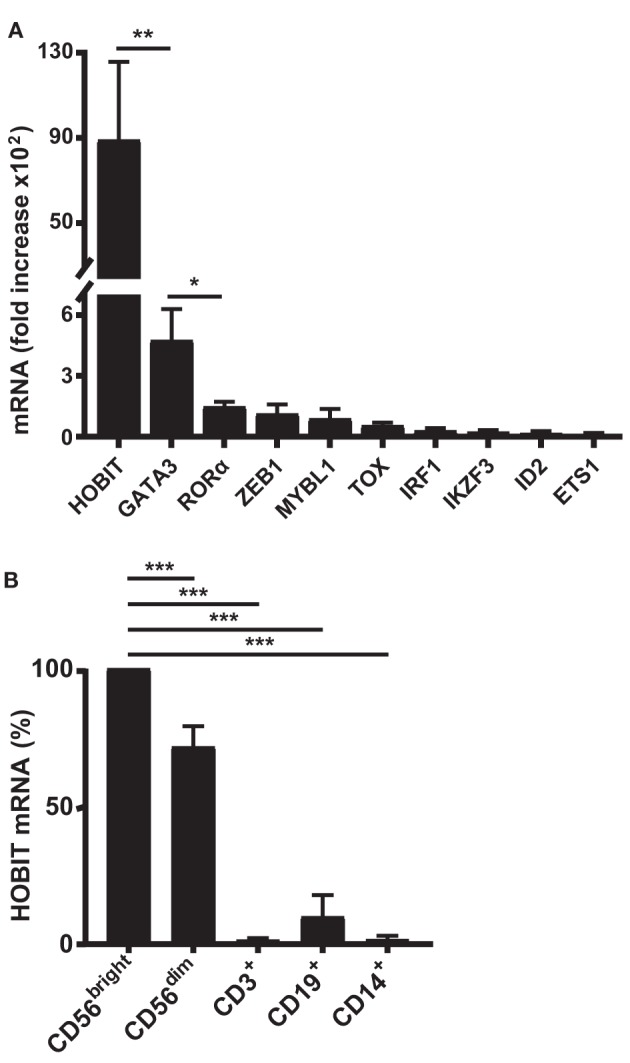
**High upregulation of ZNF683/HOBIT mRNA during *ex vivo* differentiation of human natural killer (NK) cells correlates with preferential expression in peripheral blood NK cells**. **(A)** Ten most highly upregulated transcription factor mRNAs during *ex vivo* NK cell differentiation: CD34^+^ stem cells from cord blood were expanded and *ex vivo* differentiated into NK cells. Cells were sampled at day 10, just before the differentiation into NK cells was initiated by the addition of IL-15, and at day 35, after 25 days of differentiation. RNA was isolated and subjected to real-time RT-PCR analysis. β-actin was used as internal control. Fold upregulation of specific mRNAs at day 35 compared to day 10 is shown. Results were calculated from three series of experiments performed in triplicates with cells from different donors and are displayed as mean ± SEM. **(B)** High expression of ZNF683/HOBIT mRNA in peripheral NK cells: mononuclear cells were isolated from human peripheral blood and one half of the cells used for isolation of NK cells by negative magnetic sorting. The NK cell fraction was further separated by flow cytometry into CD56^bright^ and CD56^dim^ NK cells. The second half of the mononuclear fraction was used to isolate CD3^+^ T lymphocytes, CD19^+^ B lymphocytes, and CD14^+^ monocytes by flow cytometry. RNA was isolated from the different cell samples and subjected to real-time RT-PCR analysis using β-actin as internal control. ZNF683/HOBIT mRNA levels within the different cell types are compared to the levels in the CD56^bright^ NK cells set to 100%. Results were obtained from three independent experiments using three different donors and are displayed as mean ± SEM (*p < 0.05, **p < 0.01, ***p < 0.001).

Initially, we were interested to identify novel transcription factors potentially contributing to human NK-cell differentiation that have not been described in this function before. For this purpose, we employed a recently developed *ex vivo* differentiation system (29). In this system, cord blood CD34^+^ stem cells are initially expanded for 10 days, prior to addition of IL-15. Following further addition of IL-2 from day 14, the differentiating cells are cultured for a total period of 35–42 days, by which time a regular culture comprises over 95% NK cells. To determine the repertoire of transcription factors differentially expressed during NK-cell development, we first performed a transcriptomic profiling study comparing samples from different time points up to day 35 with cultures at day 10 (start of the NK-cell differentiation). The 20 most highly upregulated transcription factor genes detected are shown in Table S1 in Supplementary Material. When real-time RT-PCR was performed for the corresponding transcripts, ZNF683/HOBIT mRNA was found to be by far the most highly upregulated mRNA at day 35 in relative terms (about 9,000-fold; Figure 1A). Among the most differentially expressed genes are many that have previously been reported to be important in NK-cell development and/or maturation such as *GATA3* (33), TOX (34), *ID2* (10), and *ETS1* (16) (Figure 1A). Based on its near absence in the stem cell cultures before addition of IL-15 and its high upregulation during differentiation, we decided to focus our further studies on ZNF683/HOBIT, especially as the role of this transcription factor in NK-cell development has not previously been reported.

### Human Peripheral Blood NK Cells Preferentially Express ZNF683/HOBIT mRNA

To determine if *ZNF683/HOBIT* is also highly expressed in adult peripheral blood NK cells, we performed real-time RT-PCR on different peripheral blood leukocyte subsets of multiple donors. Indeed, compared to T and B lymphocytes as well as monocytes, ZNF683/HOBIT mRNA is predominantly expressed in NK cells (Figure 1B). CD19^+^ B lymphocytes display lower levels of ZNF683/HOBIT mRNA (about 7-fold less as compared to CD56^dim^ NK cells) and the levels in T lymphocytes and monocytes are even much lower or at detection limit. We were furthermore interested to compare the ZNF683/HOBIT mRNA levels between the more immature CD56^bright^ and the rather mature CD56^dim^ subpopulations of NK cells. The data show that CD56^bright^ cells express the highest levels of ZNF683/HOBIT mRNA, whereas CD56^dim^ cells display about 20% lower levels (Figure 1B).

### ZNF683/HOBIT mRNA Is Upregulated in Parallel to the Generation of NK Cells

**Figure 2:**
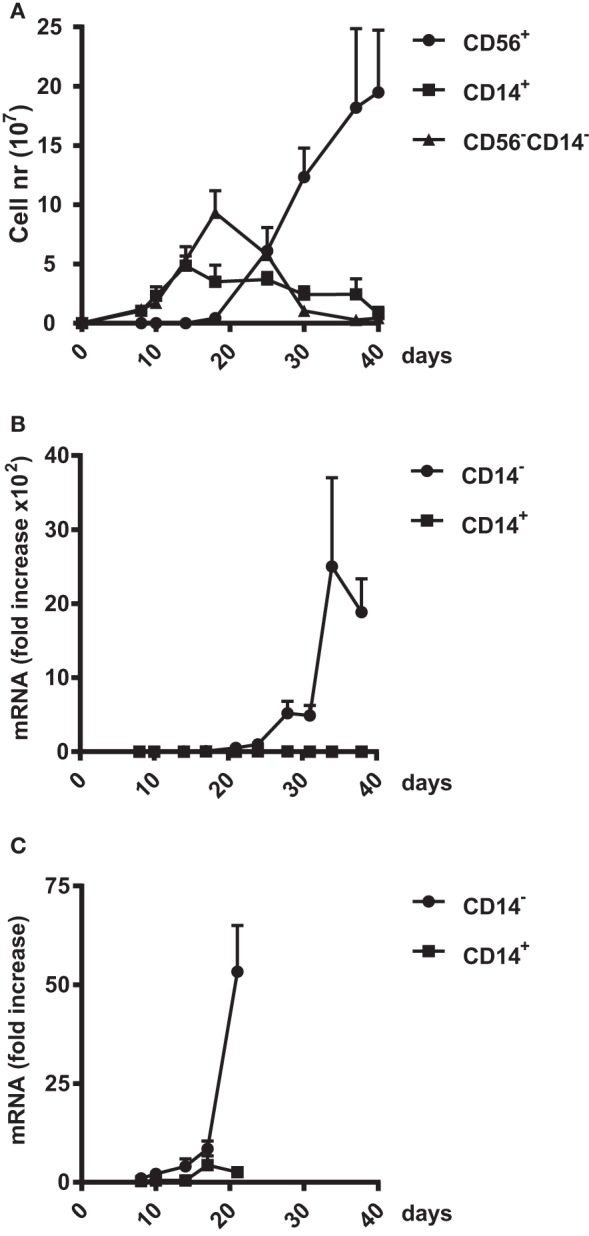
**Upregulation of ZNF683/HOBIT mRNA during natural killer (NK) cell differentiation**. **(A)** Amplification of three different cell populations in the *ex vivo* NK-cell differentiation cultures. Cord blood CD34^+^ cells were differentiated into NK cells over a culture period of 40 days. In regular intervals, cells were analyzed by flow cytometry for expression of the monocytic marker CD14 and the NK cell marker CD56. The numbers of CD56^−^CD14^−^, CD56^+^CD14^−^, and CD56^−^CD14^+^ cells were plotted. Results are calculated from 10 independent experiments using cells of different donors and are displayed as mean ± SEM. **(B,C)** Upregulation of ZNF683/HOBIT mRNA levels. Cell samples were taken at the indicated time points and CD14^+^ cells separated from the CD14^−^ population using magnetic sorting. RNA was isolated and subjected to real-time RT-PCR analysis with β-actin as internal control. Results are calculated from three independent series of experiments performed in triplicates using different donors. Fold upregulation in comparison to the values obtained for day 8 cells is shown as mean ± SEM **(B)**. To display early ZNF683/HOBIT mRNA upregulation the period until day 21 is shown at a larger scale **(C)**.

To define the potential role(s) of ZNF683/HOBIT throughout NK-cell development, we evaluated the kinetics of accumulation of ZNF683/HOBIT mRNA during *ex vivo* differentiation. During this process, three different cell types can be distinguished based upon the monocytic and NK-cell markers, CD14 and CD56, respectively (28). One of the subpopulations arising from the amplifying CD34^+^ stem cells consists of monocytic CD14^+^ cells that accumulate until day 14 after which they gradually disappear (Figure 2A). The second population consists of CD56^−^CD14^−^ cells, which peak at day 18. These CD56^−^CD14^−^ cells are presumed to comprise at least in part the NK-cell progenitors as indicated by their rapid decrease from day 18 onward, along with the concomitant rapid generation of CD56^+^ NK cells (Figure 2A). The CD56^+^ NK cells become the predominant population after day 25 and from day 35 onward the culture consists of over 95% CD56^+^ NK cells (Figure 2A).

We obtained samples at different time points during the *ex vivo* cultures and separated CD14^−^ and CD14^+^ cells by flow cytometry. We detected that ZNF683/HOBIT mRNA is nearly absent after the initial expansion phase of 10 days. A tremendous accumulation of ZNF683/HOBIT mRNA is observed from day 24 onward in CD14^−^ cells comprising NKPs and CD56^+^ NK cells (Figure 2B). The magnitude of ZNF683/HOBIT mRNA accumulation actually blurs the onset of expression in the display of Figure 2B. When plotted at a higher magnification, it is apparent that ZNF683/HOBIT mRNA increased in CD14^−^ cells already until day 17 followed by a first exponential accumulation up to day 21 (Figure 2C). Although we observed low expression of ZNF683/HOBIT mRNA in the CD14^+^ monocytes at day 17, the following first accumulation is absent (Figure 2C) as well as the exponential increase later during differentiation (Figure 2B).

### ZNF683/HOBIT Downmodulation Substantially Reduces Expansion of NK Progenitors

**Figure 3:**
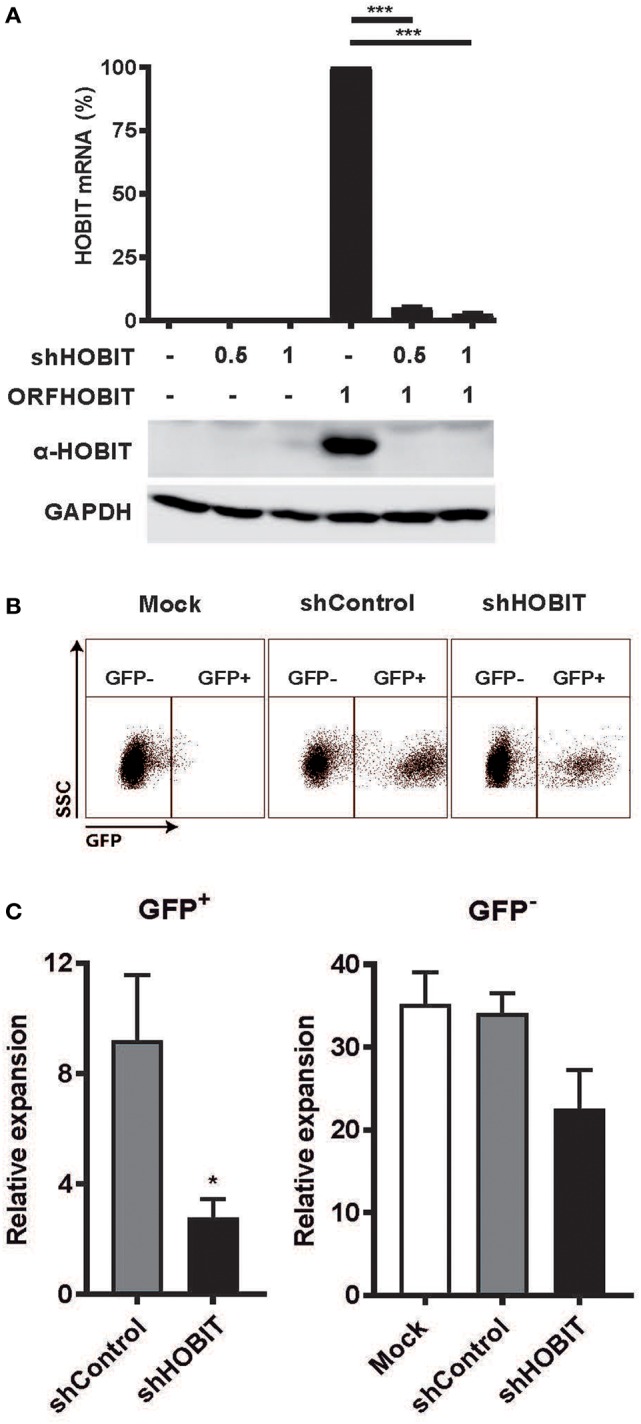
**Effects of transduction with lentiviruses expressing HOBIT shRNA on total cell expansion**. **(A)** HOBIT shRNA strongly reduces HOBIT expression. Lentiviral vectors expressing HOBIT shRNA were co-transfected with a HOBIT expression construct into HEK293T cells. After 96 h, cells were either used for RNA isolation or lysed in Laemmli sample buffer. The RNA was used for realtime RT-PCR analysis (upper part). Results are derived from two experiments performed in quadruplicates and shown as mean ± SD. The proteins in the lysed samples were separated by SDS-PAGE, Western blotted, and probed with anti-HOBIT antibodies (lower part). As internal control hypoxanthine-guanine phosphoribosyltransferase was detected by respective antibodies. Two experiments with comparable results were performed. **(B,C)** HOBIT shRNA reduces expansion of cells at day 21. Cord blood CD34^+^ cells were cultured for 5 days, then cells were transduced with lentiviruses expressing either shHOBIT or a scrambled control shRNA (shControl) or were mock-treated. Transduction efficiency was measured 3 days later by flow cytometry scoring GFP-positive cells. Cells were further cultured and differentiated until day 21. Then flow cytometry was performed to evaluate expansion of transduced GFP-expressing cells. Exemplary dot plots for cells transduced with lentiviruses expressing shHOBIT or shControl or mock-transduced controls are shown in **(B)**. The numbers of the transduced GFP^+^ cells and the non-tranduced GFP^−^ cells in individual cultures was calculated from the measured cell number and the respective percentages determined by flow cytometry and are shown in **(C)**. The values were normalized to the number of transduced or non-tranduced cells measured at day 8 to establish the expansion rates. Results are calculated from four experiments performed in triplicates and are displayed as mean ± SEM (*p < 0.05, ***p < 0.001).

After observing that ZNF683/HOBIT mRNA is mainly present in NK cells and their progenitors, we set out to elucidate the potential role(s) of ZNF683/HOBIT during NK-cell development and maturation in more detail. For this purpose, we performed loss-of-function studies using a lentiviral-based shRNA-mediated knockdown of ZNF683/HOBIT. The appropriate functioning of the shRNA was confirmed by shRNA-mediated knockdown of ectopically expressed ZNF683/HOBIT in HEK293T cells. As shown in Figure 3A, expression of ZNF683/HOBIT mRNA and protein was strongly reduced (>90%) upon co-expression of the shRNA, thus confirming the proper functioning of the shRNA (Figure 3A).

We then continued to investigate the effects of ZNF683/HOBIT knockdown on *ex vivo* differentiation cultures. These cultures were transduced with lentiviruses expressing either ZNF683/HOBIT shRNA (shHOBIT) or a scrambled control shRNA (shControl). Cultures, mock-treated for the transduction procedure but without addition of viruses, were performed in parallel. Based on initial experiments that evaluated best transduction rates and survival in relation to days in culture, cells were transduced during the stem cell expansion phase 5 days after isolation. The presence of a GFP cassette driven by a spleen focus-forming virus (SFFV)-promoter enabled us to discriminate transduced GFP^+^ cells expressing the shRNA from non-transduced GFP^−^ cells within the same culture in parallel (35). Regularly, transduction rates between 30 and 50% were achieved. Since these varied to some extent, we normalized obtained expansion rates for the analyzed fractions to the different transduction rates measured 3 days after transduction (8 days after isolation).

Based on the fact that the expression of the NK-cell marker CD56 becomes significant only from day 24 onward, we decided to split the analysis in two parts. In the first part, we analyzed the NKP stage until day 21, in the second part the generation and maturation of CD56^+^ NK cells. At day 21, we observed a fourfold reduced expansion of the shHOBIT GFP^+^ cells compared to the shControl GFP^+^ cells (Figure 3C). Exemplary dot plots of the GFP staining are shown in Figure 3B. The GFP^−^ cells of the shHOBIT culture also seemed to be somewhat reduced in comparison to the scrambled control and regular mock cultures, but the difference did not reach statistical significance.

**Figure 4:**
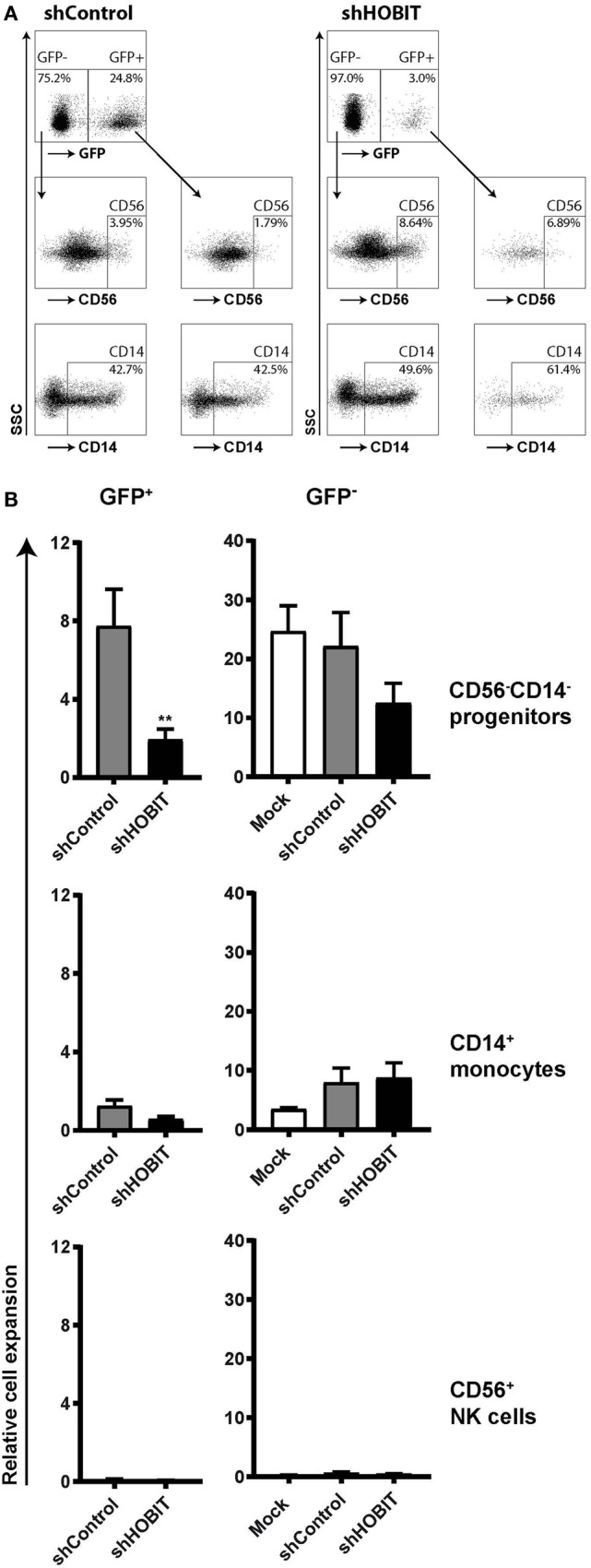
**HOBIT shRNA strongly reduces the number of CD56^−^CD14^−^ progenitor cells at day 21**. Stem cells at day 5 of culture were transduced and further cultured until day 21. Then CD56 and CD14 expression was assessed by flow cytometry within the GFP^+^ and GFP^−^ parts of the culture. **(A)** Exemplary flow cytometry dot plots displaying staining for CD56 and CD14 at day 21. **(B)** The relative expansion of CD56^−^CD14^−^, CD56^+^, and CD14^+^ cells within the GFP^+^ and GFP^−^ fractions is shown. Expansion rates were calculated from the measured cell numbers at day 21 and the percentages of the different subsets obtained by flow cytometry. Differences in transduction efficiency were corrected for by normalizing the obtained values to the number of GFP^+^ and GFP^−^ cells at day 8, respectively. Results are calculated from four independent experiments performed in triplicates using different donor cells and are displayed as mean ± SEM (**p < 0.01).

Since at day 21 the culture comprises mainly two different cell types, CD56^−^CD14^−^ progenitor cells and CD14^+^ monocytic cells (Figure 2A), we continued to analyze these populations separately. Exemplary dot plots of this analysis are displayed in Figure 4A. The data showed that the effect can be mainly traced to a fourfold reduced expansion of the CD56^−^CD14^−^ progenitors in the shHOBIT GFP^+^ fraction. Although smaller inhibitory effects were also visible for CD14^+^ cells of the shHOBIT GFP^+^ pool and the GFP^−^ cells of the shHOBIT culture, both did not reach statistical significance (Figure 4B). However, this may indicate that a strong inhibitory effect on a fraction of the culture indirectly affects the whole culture, including the GFP^−^ part.

The number of CD56^+^ NK cells was too low to be analyzed in detail at this time point of the culture (Figures 4A,B). Together, the data clearly showed that knockdown of ZNF683/HOBIT reduces the expansion of presumptive NKPs already at day 21 of the culture. This suggests a role of ZNF683/HOBIT in the early stages of human NK-cell development before the initiation of CD56 expression.

### ZNF683/HOBIT Downmodulation Largely Abrogates the Generation of CD56^+^ NK Cells

**Figure 5:**
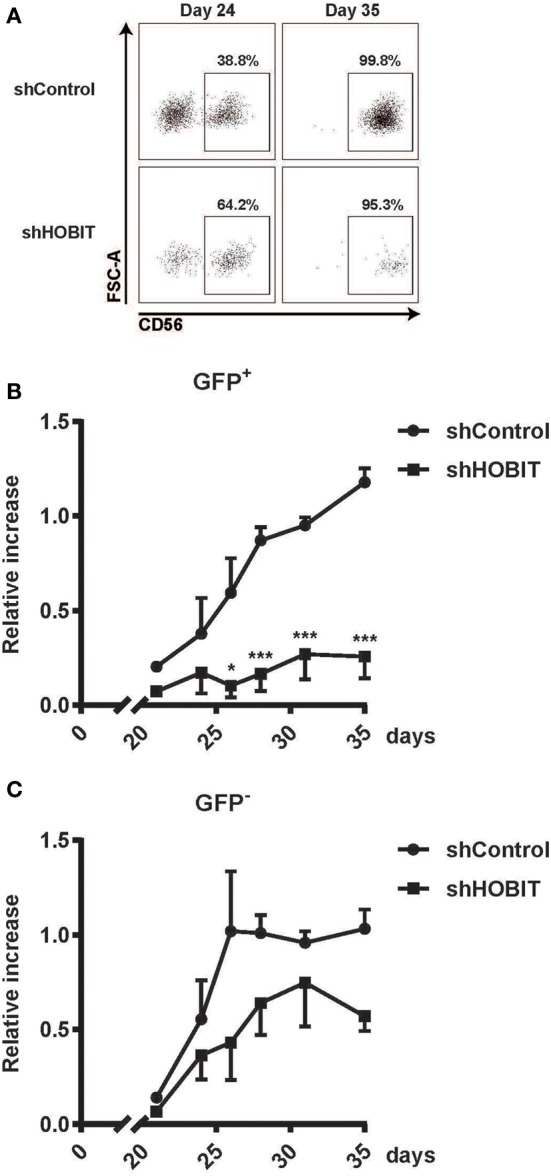
**HOBIT shRNA nearly abrogates formation of CD56^+^ natural killer (NK) cells**. Stem cell cultures were transduced and cultured until day 35. The generation of CD56^+^ NK cells was monitored by flow cytometry in samples taken at days 24, 26, 28, 31 and 35. **(A)** Exemplary flow cytometry dot plots displaying staining for CD56 within the GFP^+^ fractions at days 24 and 35. **(B)** The formation of CD56^+^ cells within the transduced GFP^+^ and non-transduced GFP^−^ fractions is shown for shHOBIT and shControl cultures. Expansion rates are calculated from measured cell numbers at the respective days and the flow cytometry-derived percentages. Differences in transduction efficiency were corrected for by normalizing the obtained values to the number of GFP^+^ and GFP^−^ cells at day 8, respectively. Results are calculated form four independent experiments, two of them performed in triplicates, using different donor cells and are displayed as mean ± SEM (*p < 0.05, ***p < 0.001).

The second part of our analysis focused on the formation of CD56^+^ cells and their maturation from day 21 onward. At first sight, when we analyzed the percentages of CD56^+^ NK cells formed during later stages at day 35, both GFP^+^ populations (shHOBIT and shControl) seemed comparable to regular cultures (>95% CD56^+^ NK cells, Figure S1 in Supplementary Material). However, when the number of cells was taken into account, it became apparent that the generation of CD56^+^ NK cells from the GFP^+^ progenitors in the shHOBIT pool is substantially reduced compared to the GFP^+^ control shRNA fraction. Whereas the GFP^+^CD56^+^ NK cells in the control shRNA cultures showed a strong expansion between days 21 and 35, the GFP^+^CD56^+^ NK cells in the shHOBIT cultures displayed almost no increase beyond day 21 and remained low in number (around 20% of the shControl cultures). These cells did not enter the typical expansion phase between days 24 and 35 (Figures 5A,B). In contrast, the GFP^−^ cells in the shHOBIT cultures displayed an expansion phase between days 21 and 30, although this expansion seemed reduced in comparison to the shControl cultures. Again this may suggest an overall indirect effect of the strongly compromised GFP^+^ cells on the GFP^−^ part of the cultures.

Taken together, our data showed that knockdown of ZNF683/HOBIT strongly abrogates the generation of CD56^+^ NK cells. This suggests that ZNF683/HOBIT downmodulation strongly affects NK-cell progenitors shortly before the initiation of CD56 expression and prevents the progression toward CD56^+^ NK cells and/or the further proliferation of the few detected CD56^+^ NK cells.

### The Few Developed CD56^+^ NK Cells Display Normal NKG2A and KIR Levels

**Figure 6:**
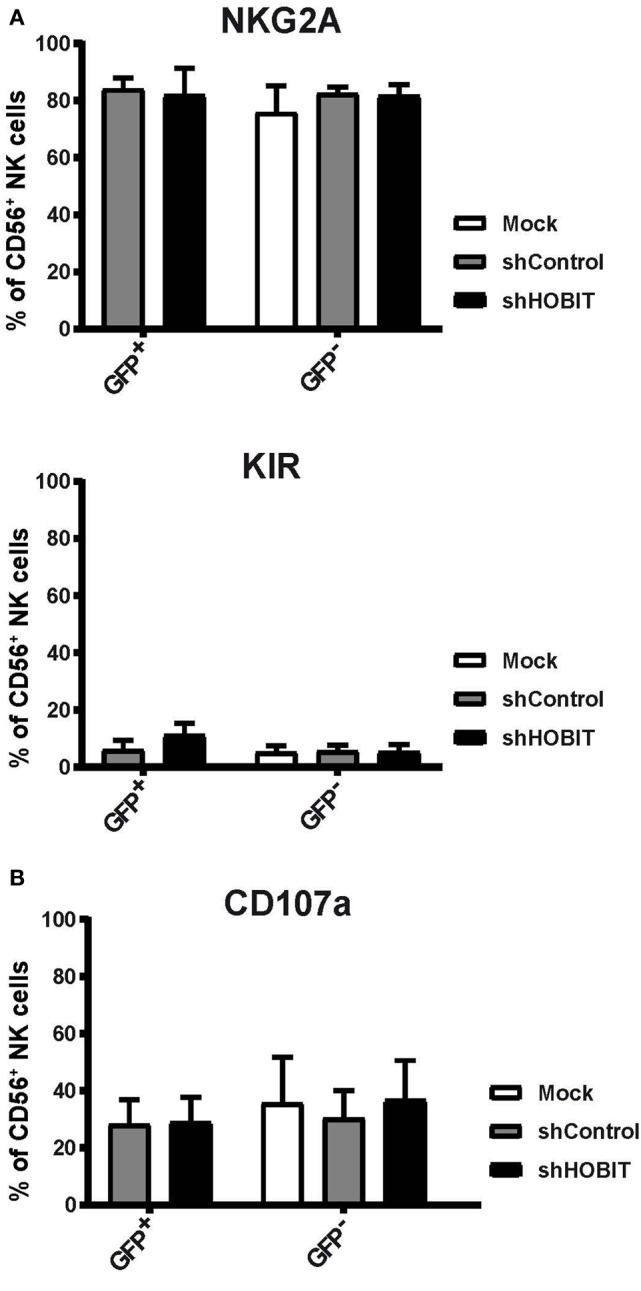

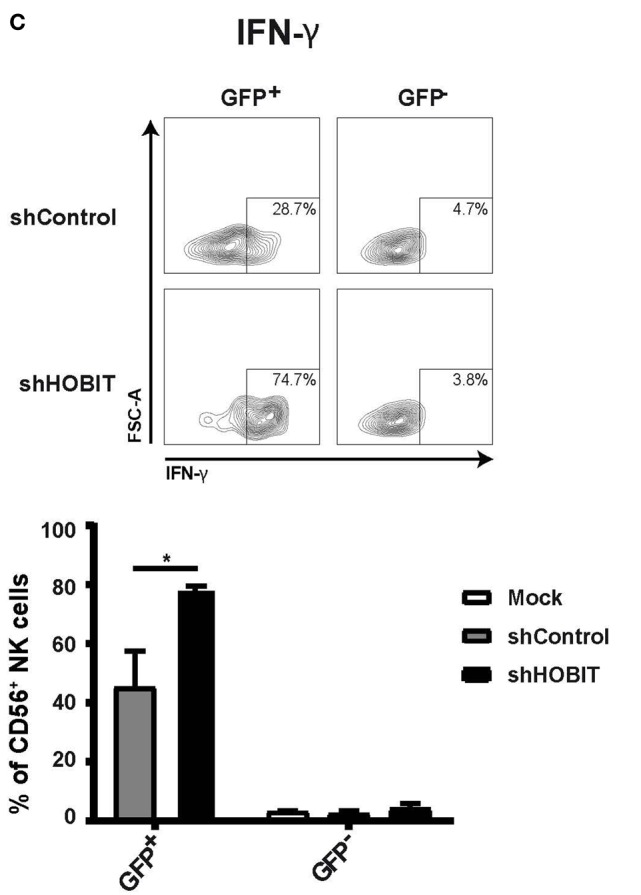
**Effects of HOBIT shRNA on phenotype and functions of natural killer (NK) cells at day 35**. **(A)** Expression of NKG2A/CD94 and KIR receptors. Stem cells were transduced and cultured until day 35. Then expression of NKG2A and KIR was measured by flow cytometry. The percentages of cells within the GFP^+^ and GFP^−^ fractions as well as in the mock-treated cultures are displayed. **(B)** Degranulation capacity: separate aliquots of the day 35 cultures were cocultured with K562 target cells and surface CD107a measured after 6 h by flow cytometry. Percentages of CD107a-positive cells within the CD56 population are displayed. **(C)** IFN-γ production: the K562 cocultures were further stained for intracellular IFN-γ. Representative flow cytometry dot plots obtained are shown in the upper part. Percentages of IFN-γ positive cells within the CD56 population are displayed in the lower part. All results are derived from four independent experiments, two of them performed in triplicates, using different donor cells and are shown as mean ± SEM (*p < 0.05).

We were further interested in the phenotype of the few detected CD56^+^ NK cells. To this end, we evaluated if these cells would further differentiate/mature *via* upregulation of NKG2A and KIR. The expression of these important NK-cell receptors was measured after 35 days of cultivation and compared between the GFP^+^ cells of the shHOBIT and the shControl cultures. We observed high levels of NKG2A in all of the GFP^+^ cells of the cultures independent of shHOBIT or control shRNA expression (Figure 6A, exemplary dot plots in Figure S2 in Supplementary Material). The GFP^−^ fractions also displayed high NKG2A levels similar to regular control cultures. Similarly, KIR expression was not significantly influenced by the presence or absence of shHOBIT or control shRNA (Figure 6A, exemplary dot plots in Figure S2 in Supplementary Material) and was comparable to the typical levels of regular cultures. In this regard, our group previously showed that *ex vivo* generated NK cells express lower levels of KIR than peripheral blood-NK cells (28). Together, these observations demonstrate that cells starting to express CD56 continue to develop normally in regard of NKG2A and KIR expression. Furthermore, it implies ZNF683/HOBIT does not influence the expression of NKG2A or KIR receptors on CD56^+^ NK cells.

### Degranulation Capacities Remain Unchanged, but IFN-γ Production Is Strongly Induced Upon Virus Transduction and Further Enhanced by ZNF683/HOBIT Knockdown

Two main functions of NK cells are cytotoxicity, the direct killing of target cells, and IFN-γ production (1, 36). To evaluate whether the CD56^+^ NK cells differentiating under knockdown conditions for ZNF683/HOBIT would functionally mature, we tested degranulation capacity in the CD107a assay and IFN-γ production. Following coculture with the target cell-line K562, we detected similar levels of CD107a on the cell surface of all cells irrespective of the culture conditions (Figure 6B, exemplary dot plots in Figure S2 in Supplementary Material). In the same cultures, we also determined the proportion of cells with intracellular IFN-γ. Intriguingly, the transduction with the scrambled control shRNA already caused a substantial increase in IFN-γ producing cells (up to 50%). This was further enhanced upon expression of the ZNF683/HOBIT shRNA leading to 80% IFN-γ-producing cells (Figure 6C). In contrast, only a very small fraction of the GFP^−^ cells was competent to produce IFN-γ (Figure 6C). In summary, transduction with shRNA lentiviruses by itself leads to a higher proportion of NK cells with the capacity to produce IFN-γ and this is further increased by knockdown of ZNF683/HOBIT. The ZNF683/HOBIT shRNA effect is restricted to an increase in the number of IFN-γ-producing cells and not reflected in the level of IFN-γ per cell as measured by mean flourescence intensity (MFI). However, virus transduced cells displayed higher levels of IFN-γ per cell in comparison to non-transduced cells (Figure S3 in Supplementary Material).

## Discussion

The recent decade has witnessed an enormous increase in the understanding of the development of different immune-cell lineages including the various forms of innate and adaptive lymphocytes from HSCs (37). Most of this knowledge is derived from the murine system due to the possibility of genetic manipulation of the animal models. Therefore, detailed knowledge on the specificities of the equivalent human differentiation and maturation pathways and factors are still elusive. Although many factors may function in identical or similar ways in murine and human immune-cell development, certain differences are to be expected based on a much more rapid evolution of the immune system when compared to other tissues. For example, this has led to the convergent evolution of distinct classes of important NK-cell receptors with similar function but encoded by structurally different genes such as the human KIR and murine Ly49 receptors (38, 39). It is conceivable that this is also reflected in the transcription factor circuitry defining human and murine NK cells.

Due to the recent increasing interest in NK cells as therapeutic agents, especially for the treatment of leukemia and potentially also solid cancers (40, 41), a more complete understanding of the control of human NK-cell differentiation and maturation is desirable. Here, we used a feeder cell-free *ex vivo* system for the generation of human NK cells from cord blood HSCs (29). This system can generate therapeutic NK cells that have been proven to be safe in a phase I clinical trial (42). We have, furthermore, demonstrated that the NK cells formed using this system display the typical NK cell receptors, potent ADCC and produce IFN-γ similar to peripheral blood NK cells (28). So this provided an ideal system to study transcription factors during human NK-cell differentiation and maturation.

Identity of cell types and their differentiation and maturation is controlled in large part by the action of transcription factors. Normally, important core transcription factors can be identified by the characteristics of upregulation during differentiation and their high expression in a relatively high cell type-specific fashion (43). In this regard, our transcriptomic profiling has identified a large number of transcription factors strongly upregulated during human NK-cell differentiation. Many of these have previously been described in the context of murine NK-cell development. For example, in earlier stages of NK-cell differentiation, ID2 and ID3 contribute via suppression of E proteins, such as E2A, the B-cell promoting factor. GATA3, TOX, and IKZF3 (AIOLOS) have been reported to promote later maturation stages of NK-cell development (20, 44). From these TOX has also been described specifically to contribute to human NK-cell differentiation, where it seems to control T-BET expression (21, 22).

Upon re-evaluation of the 10 most highly upregulated transcription factor genes from the profiling experiment, it was apparent that ZNF683/HOBIT mRNA was the most strongly upregulated. It displayed a 10-fold higher upregulation compared to GATA-3, the second best factor. Further analysis of different immune-cell types in human peripheral blood showed preferential expression of ZNF683/HOBIT mRNA in CD56^+^ NK cells when compared to B and T lymphocytes and monocytes. We detected low expression levels in B cells, but it was very low to undetectable in T lymphocytes and monocytes. Furthermore, *ZNF683* is a homolog of the *PRDM1* gene encoding BLIMP-1 (ZNF683 has, therefore, also been termed HOBIT, homolog of Blimp-1 in T cells) (45). In the mouse, Blimp-1 has been shown to be a master regulator of terminal differentiation of CD8^+^ effector T cells and plasma cells (46) and to play a role in the maturation of peripheral NK cells (18). Some upregulation of *PRDM1* was detected in our profiling analysis (Table S1 in Supplementary Material), but BLIMP-1 mRNA showed a much less dramatic increase during human NK-cell differentiation than ZNF683/HOBIT mRNA (Table S2 in Supplementary Material). Considering its selective upregulation and expression and its homology with BLIMP-1, an established important differentiation factor, ZNF683/HOBIT appeared to fulfill the precondition for a new key transcription factor controlling the development of human NK cells.

We further analysed ZNF683/HOBIT expression in the classical defined NK subsets, CD56^bright^ and CD56^dim^ cells. It is thought that the CD56^bright^ cells are more immature, preferentially produce cytokines, and will mature further into CD56^dim^ cells that display potent cytotoxic activity (1). In our analysis, evaluation of ZNF683/HOBIT mRNA revealed high expression levels in both subsets with somewhat higher levels in CD56^bright^ cells. Together with the kinetic analysis of ZNF683/HOBIT mRNA accumulation, that displayed a parallel increase with the generation of CD56^+^ NK cells, this initially suggested a preferential activity between acquisition of CD56 and the CD56^bright^ cell stage. However, a closer inspection revealed significantly increased ZNF683/HOBIT mRNA levels already in day 14-progenitors, with an exponential upregulation from day 18 onward. This suggests that ZNF683/HOBIT mRNA starts to be expressed soon after addition of IL-15 in NKPs and continuously increases with highest accumulation rates in parallel to the increase in CD56 expression. In comparison, only minor ZNF683/HOBIT mRNA levels appeared to be present in the CD14^+^ monocytic subset, which develops between days 10 and 20 and disappears thereafter.

In line with the early expression of ZNF683/HOBIT at the NKP stage, shRNA-mediated downmodulation of ZNF683/HOBIT resulted in a significantly reduced expansion of transduced cells already at day 21 of the culture. The majority of this effect could be traced to the CD56^−^CD14^−^ cells containing at least in part the NKPs. CD56^+^-expressing NK cells were not yet detectable at significant amounts at this time point in the culture. This clearly supports that a first major effect of ZNF683/HOBIT downmodulation is a reduction of proliferation and/or survival of NK-cell progenitors prior to the initiation of CD56 expression. Some reduction seemed also to occur for the CD14^+^ cells in shHOBIT transduced cultures, although this did not reach significance. As very low levels of ZNF683/HOBIT mRNA appear to be expressed in CD14^+^ cells, we are unable to rule out a potential specific negative effect also upon this subset following HOBIT knockdown.

The second major effect became apparent when we monitored the acquisition of CD56 expression. CD56 or N-CAM is generally accepted as a major marker for human NK cells, despite that its function remains elusive (47). It is present on about 95% of human NK cells (47), but lacks a clear homolog in murine NK cells (48). The kinetics of accumulation of CD56^−^CD14^−^ progenitors and CD56^+^ cells in the *ex vivo* system are compatible with the start of expression of CD56 by CD56^−^CD14^−^ progenitors from day 20 onward. This is indicated by the rapid decline of CD56^−^CD14^−^ progenitors and the concomitant appearance of the CD56^+^ cells. This generation of CD56^+^ cells was nearly abrogated upon downmodulation of ZNF683/HOBIT. This supports that ZNF683/HOBIT is essential for efficient generation of CD56 expressing cells and/or their further proliferation or survival.

Important receptors in human NK cells are the NKG2A and KIR receptors (11, 49). In the *ex vivo* cultures, the expression of NKG2A starts shortly after CD56 expression (28). NKG2A is further expressed on the majority of the more immature CD56^bright^ cells. According to the current hypothesis, when these cells mature into CD56^dim^ cells, they will reduce NKG2A and increase KIR expression (11). Despite the substantially reduced number of CD56^+^ cells formed in the shHOBIT cultures, it was astonishing that these cells appeared quite normal in respect of NKG2A expression compared to control cultures. The observed levels of NKG2A expression on about 80% of CD56^+^ cells were comparable to the levels characteristic for regular cultures (28). Also KIR levels established with a pan-KIR antibody were similar to regular cultures, in the order of 5% of CD56^+^ cells. As previously discussed (28), in the *ex vivo* cultures NKG2A levels are similarly high as in peripheral CD56^bright^ cells, probably due to the high cytokine levels in the culture. KIR levels are intermediary between CD56^bright^ cells and CD56^dim^ cells. This suggests that *ex vivo*-generated NK cells, in terms of receptor expression, do not fully mature, whereas functionally they display full cytotoxic capabilities. In this regard, we observed no differences in degranulation capabilities against K562 targets between the shHOBIT and control cultures.

In contrast, it was remarkable that virus transduction by itself caused an increase in the proportion of IFN-γ-producing CD56^+^ NK cells. About 50% of day 35 cells transduced with control shRNA viruses produced IFN-γ upon coculture with K562 target cells, whereas only a few percent for non-transduced cells. Currently, it is unclear whether this is due to a priming process at the time of transduction at day 5 that continues to act into later stages of differentiation or whether it is high expression of shRNAs that can activate the differentiated cells. We can only speculate about the underlying mechanisms for this unexpected finding. For example, it has been described that a uridine-rich part of the HIV RNA (ssRNA40) can activate NK cells *via* TLR7/8 signaling (50). So, maybe the presence of high levels of shRNA or RNA transcribed from the viral sequences could somehow activate the IFN-γ machinery. Irrespective of the cause of this effect, the increase in IFN-γ-producing cells initiated by the virus itself was further enhanced by knockdown of ZNF683/HOBIT. This suggests that ZNF683/HOBIT negatively controls the development of IFN-γ-producing NK cells.

We have to introduce a caveat on the interpretation of the data for the late stages of differentiation in the *ex vivo* sytem. Due to the low numbers of GFP^+^ cells obtained, that further appeared to be too fragile for preparative sorting, we were unable to exclude that the few late stage GFP^+^ NK cells generated are derivatives of few cells that failed to express the shRNA and, therefore, continued to differentiate. It is, however, equally conceivable that ZNF683/HOBIT knockdown is effective on proliferation and/or survival only within a certain time window, when the cells normally still proliferate, and is less effective once the cells have passed this stage and do not proliferate but rather further differentiate and mature.

While this work was in progress, a report on ZNF683/HOBIT was published describing that the factor is highly expressed in human effector-type CD8^+^ T cells, but not in naive or most memory CD8^+^ T cells or CD4^+^ helper T cells (51, 52). High levels of ZNF683/HOBIT were expressed in CMV-specific, but not in influenza-specific CD8^+^ T cells. This may explain why in our experiments we did not detect significant expression levels in peripheral T cells as we only tested the overall population of CD3^+^ T cells comprising all CD4^+^ and CD8^+^ T cells. This report also showed highest expression in human NK cells and low expression in dendritic cells in accordance with our data on peripheral NK cells and monocytes.

Currently available evidence suggests that ZNF683/HOBIT may be special in terms of displaying substantial differences in expression pattern and possibly function in different cell types in mice and humans. In mice, it was reported to be predominantly expressed in NKT cells, a CD4^+^ T cell subset with immediate effector functions, and to some extent in CD8^+^ T cells (45, 53). In humans, ZNF683/HOBIT is mainly expressed in NK cells and, as described by others, also in effector-type CD8^+^ T cells (51). Differences also seem to occur regarding the control of IFN-γ production. In murine NKT cells, ZNF683/HOBIT represses IFN-γ and activates granzyme B production (45). In contrast, in human long-lived effector T cells, ZNF683/HOBIT was reported to induce IFN-γ and to have no effect on granzyme B production (51). Our findings in human NK cells rather support a suppressing effect of ZNF683/HOBIT on IFN-γ production. This correlates with a report on BLIMP-1 describing a similar suppression of IFN-γ production in human NK cells (51). No effect of BLIMP-1 on cytotoxicity was observed, which is also in agreement with our findings on ZNF683/HOBIT. Generally, the homology of ZNF683/HOBIT and BLIMP-1 suggests overlapping and synergistic activities. Both factors display highly conserved zinc finger domains mediating binding to DNA target sequences in enhancer regions of a number of genes. The DNA binding sites of the factors largely overlap and both can bind to target sequences in several identical genes including *TCF7*. Both factors seem to mainly act as transcriptional repressors (44, 54), although for BLIMP-1 activating properties have also been described (55).

Taken together, our data for the first time show a role of ZNF683/HOBIT during differentiation of human NK cells. They strongly support that ZNF683/HOBIT is a key regulatory factor controlling generation, proliferation, and/or survival of NK-cell progenitors (CD56^−^CD14^−^) and is essential for efficient generation of CD56^+^ cells. Once CD56 expression has been acquired the further maturation including NK receptor expression and development of degranulation capacity seems to be unaffected, whereas IFN-γ production appears to be constrained by ZNF683/HOBIT. Although a more precise identification of the developmental stage(s) affected will need additional investigations, the data are compatible with the possibility that ZNF683/HOBIT may mainly act between early factors, such as NFIL3 (56) with proposed activities at the early committed NKP stage and factors described to promote rather maturation of NK cells at later stages such as GATA-3 (33). It further remains to be established how this is achieved in potential interaction with BLIMP-1 described so far as important mainly for late stage maturation.

## Ethics Statement

Human umbilical cord blood samples were obtained at birth after full-term delivery from the Department of Obstetrics and Gynecology of the University Hospital of Vienna, Austria. This was carried out in accordance with the recommendations of the “Ethical committee of the Medical University of Vienna” with written informed consent from all subjects in accordance with the Declaration of Helsinki. The protocol was approved by the “Ethical committee of the Medical University of Vienna” (protocol number 122/2010).

## Author Contributions

MP performed experiments and wrote the basic manuscript. AC aided in experimental design, performed experiments, and improved the manuscript. DL provided the basis of the study with initial experiments, including the transcriptional profiling, and MO contributed with part of real-time RT-PCR analyses. UR helped with Western blots and DD with generating viral constructs. BS, WE, AN, JS, and DS contributed in the design of the study, provided cell samples, viral vectors and technology, and corrected the manuscript. EC contributed to the design, supported the progress of the project, and helped with formulating the final manuscript. EH conceived the study, supported MP and AC in all aspects of the experiments, and helped to finish the final version of the manuscript.

## Conflict of Interest Statement

One of the coauthors, JS, is CSO of Glycostem Therapeutics. This company has developed and sells GBGM medium for *ex vivo* generation of therapeutic NK cells. Glycostem Therapeutics has contributed differentiated cells in the initial phase and GBGM medium throughout the course of this work. Purchase and/or use of GBGM medium for scientific purposes is not restricted by patent rights. All of the other authors have no financial or commercial interests connected to Glycostem Therapeutics or other companies that could be construed as a potential conflict of interest.

## Footnotes

^1^Affymetrix support site; www.affymetrix.com/support/index.affx.

^2^www.bioconductor.org/.

^3^https://www.thermofisher.com/order/catalog/product/15596026?ICID=cvc-rna-cultured-cells-c1t1.

^4^http://www.sciencegateway.org/protocols/lentivirus/cloning.htm.

^5^https://www.ucl.ac.uk/cancer/research/scientific-facilities-and-services/cancer-genomics-engineering-facility/rnai-library/open-biosystems-pgipz-rna-library.
