# Supplementary material for: The Transcription Factor ZNF683/HOBIT Regulates Human NK-Cell Development
Source: Front Immunol. 2017 May 15;8:535. doi: 10.3389/fimmu.2017.00535 (PMC5430038; doi:10.3389/fimmu.2017.00535)
Supplement: Supplementary file 1 [file presentation_1.pdf]

# *Supplementary Material*

## The transcription factor ZNF683/HOBIT regulates human NK cell development

**Mirte Post, Angelica Cuapio, Markus Osl, Dorit Lehmann, Ulrike Resch, David M. Davies, Martin Bilban, Bernhard Schlechta, Wolfgang Eppel, Amit Nathwani, Dagmar Stoiber, Jan Spanholtz, Emilio Casanova and Erhard Hofer***

*** Correspondence:**
Erhard Hofer
erhard.hofer@meduniwien.ac.at
erhard.hofer@gmx.at

**Supplementary Table S1. List of the 20 genes most strongly upregulated during *ex-vivo* differentiation of NK cells.**
$CD34^+$ stem cells were isolated from one cord blood sample and *ex vivo* differentiated into NK cells as described in the methods section. Cells were collected at several time points between10 and 35 days of culture, total RNA was isolated and subjected to Affymetrix microarray analysis. Changes in gene expression are displayed as fold induction relative to day 10 of culture. Genes are listed according to decreasing induction rates. Genes listed several times in the original Affymetrix array data set are only given once using their highest value.

| | mRNA accession | *Gene symbol* | Gene full name | Day 15 | Day 17 | Day 20 | Day 22 | Day 24 | Day 27 | Day 29 | Day 31 | Day 35 |
|---|---|---|---|---|---|---|---|---|---|---|---|---|
| 1 | NM_001080416.3 | ***MYBL1*** | MYB proto-oncogene like 1 | 1.2 | 1.5 | 2.8 | 8.6 | 15.9 | 31.7 | 77.2 | 96.5 | 158.3 |
| 2 | NM_012481.4 | ***IKZF3 (Aiolos)*** | IKAROS family zinc finger 3 | 0.69 | 1.24 | 1.4 | 1.5 | 2.19 | 4.31 | 6.16 | 9.97 | 15.33 |
| 3 | NM_001143820.1 | ***ETS1*** | ETS proto-oncogene 1 | 1.46 | 1.64 | 1.69 | 2.71 | 2.71 | 4.49 | 7.53 | 9.68 | 13.75 |
| 4 | NM_003151.3 | ***STAT4*** | Signal transducer and activator of transcription 4 | 1.45 | 2.38 | 1.81 | 2.54 | 2.87 | 4.16 | 6.96 | 8.63 | 13.13 |
| 5 | NM_001002295.1 | ***GATA3*** | GATA-binding factor 3 | 0.92 | 0.91 | 1.11 | 1.15 | 1.49 | 2.02 | 3.08 | 4.11 | 9.59 |
| 6 | NM_001114759.2 | ***ZNF683*** | Zinc finger protein 683 | 0.97 | 1.04 | 1.24 | 1.20 | 1.91 | 2.77 | 2.90 | 4.30 | 8.87 |
| 7 | NM_005442.3 | ***EOMES*** | Eomesodermin | 1.07 | 1.16 | 1.58 | 1.64 | 2.32 | 4.01 | 5.22 | 5.57 | 7.93 |
| 8 | NM_014729.2 | ***TOX*** | Thymocyte associated high mobility group box | 1.32 | 1.51 | 1.27 | 1.49 | 1.86 | 2.47 | 3.68 | 5.1 | 7.73 |
| 9 | NM_134260.2 | ***RORA (RORα)*** | RAR related orphan receptor A | 1.04 | 1.25 | 0.99 | 1.19 | 1.50 | 1.68 | 2.10 | 2.27 | 4.14 |
| 10 | NM_003670.2 | ***BHLHE40*** | Basic helix-loop-helix family member e40 | 1.46 | 1.76 | 1.66 | 1.91 | 2.10 | 2.92 | 4.57 | 4.08 | 4.06 |
| 11 | NM_001323638.1 | ***ZEB1*** | Zinc finger E-box binding homeobox 1 | 0.98 | 1.28 | 1.05 | 1.13 | 1.18 | 1.71 | 2.34 | 2.46 | 3.89 |
| 12 | NM_007249.4 | ***KLF12*** | Kruppel like factor 12 | 0.80 | 1.07 | 0.90 | 0.85 | 1.09 | 1.38 | 2.01 | 1.82 | 3.86 |
| 13 | NM_001031680.2 | ***RUNX3*** | Runt related transcription factor 3 | 1.17 | 1.80 | 1.40 | 1.65 | 2.47 | 3.04 | 3.16 | 3.51 | 3.85 |
| 14 | NM_002167.4 | ***ID3*** | Inhibitor of DNA binding 3, HLH protein | 1.38 | 1.45 | 1.32 | 1.23 | 1.83 | 1.78 | 2.56 | 2.59 | 3.71 |
| 15 | NM_001198.3 | ***PRDM1*** | PR/SET domain 1 | 2.76 | 4.20 | 2.66 | 2.96 | 3.70 | 4.62 | 2.60 | 2.58 | 3.44 |
| 16 | NM_021813.3 | ***BACH2*** | BTB domain and CNC homolog 2 | 0.86 | 0.88 | 0.80 | 0.85 | 0.96 | 1.38 | 1.53 | 1.86 | 3.13 |
| 17 | NM_002166.4 | ***ID2*** | Inhibitor of DNA binding 2, HLH protein | 1.28 | 1.56 | 1.44 | 1.26 | 1.31 | 1.68 | 2.37 | 2.30 | 2.92 |
| 18 | NM_007315.3 | ***STAT1*** | Signal transducer and activator of transcription 1 | 1.10 | 1.48 | 1.41 | 1.60 | 2.31 | 4.39 | 5.66 | 4.27 | 2.77 |
| 19 | NM_002198.2 | ***IRF1*** | Interferon regulatory factor 1 | 1.26 | 1.27 | 1.59 | 1.99 | 2.07 | 3.35 | 4.53 | 3.69 | 2.75 |
| 20 | NM_002163.2 | ***IRF8*** | Interferon regulatory factor 8 | 1.89 | 1.67 | 2.21 | 2.61 | 2.74 | 2.80 | 2.67 | 2.21 | 2.45 |

**Supplementary Table S2. Comparison of the upregulation of ZNF683/HOBIT and PRDM1/BLIMP-1 mRNA during NK cell differentiation.**
RNA was isolated between days 21 and 35 and realtime RT-PCR performed. β-actin was used as internal control. Results are calculated as fold upregulation in comparison to the values obtained for day 10 cells and are shown as mean +/- SEM. Two independent series of experiments were performed in triplicates.

| | HOBIT | BLIMP-1 |
|---|---|---|
| **Day 21** | 59 (+/- 52) | 8 (+/- 4) |
| **Day 28** | 413 (+/- 254) | 10 (+/- 7) |
| **Day 35** | 1185 (+/- 615) | 18 (+/- 13) |

**Supplementary Table S3. List of Primers used for realtime RT-PCR analysis.**

| Primer | Sequence (5' - 3' direction) |
|---|---|
| HOBIT-1 Forward | CATATGTGGCAAGAGCTTTGG |
| HOBIT-1 Reverse | GGCAAGTTGAGTGAAGCTCT |
| HOBIT-2 Forward | GGTCCCATTGAGTTCCCAGA |
| HOBIT-2 Reverse | GGTGGGCAAGTTGAGTGAAG |
| BLIMP1-1 Forward | TACATACCAAAGGGCACACG |
| BLIMP1-1 Reverse | TGAAGCTCCCCTCTGGAATA |
| ß-actin Forward | TTTGAATGATGAGCCTTCGTCCCC |
| ß–actin Reverse | GGTCTCAAGTCAGTGTACAGGTAAGC |
| HPRT Forward | AAGCTTGCTGGTGAAAAGGA |
| HPRT Reverse | CAAACATGATTCAAATCCCTGA |

**Supplementary Table S4. Monoclonal antibodies used for flow cytometry analysis.**

| CD number | Alternative name | Fluoro-chrome | Clone | Vendor | Cat. No |
|---|---|---|---|---|---|
| **CD56** | N-CAM | PeCy7 | B159 | BD | 557747 |
| **CD14** | | PerCPCy5.5 | MφP9 | BD | 562692 |
| **CD159a** | NKG2A | APC | Z199 | BC | A60797 |
| **CD158** | KIR | PE | 180704 | RD | FAB1848P |
| **CD107a** | Lysosomal-associated membrane protein 1 (LAMP-1) | APC | H4A3 | BD | 560664 |
| **IFN-γ** | | PE | 4S.B3 | BD | 559326 |
| **CD34** | | APC | AC136 | Miltenyi | 130-098-139 |

*BD = BD Biosciences; BC = Beckman Coulter; RD = R&D Systems*

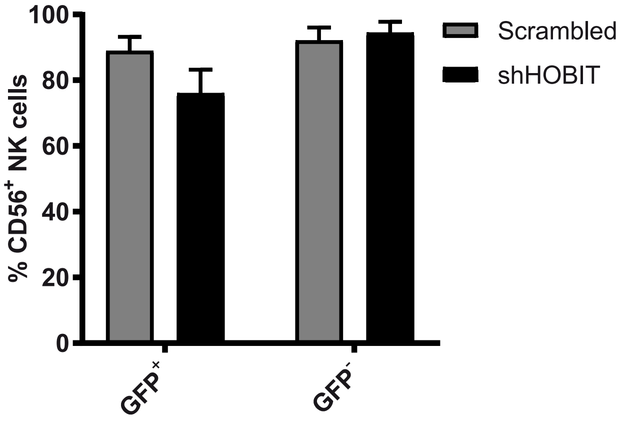


**Supplementary Figure 1. Effects of HOBIT shRNA on the percentage of CD56+ cells at day 35.** Cord blood CD34+ stem cells expanded for 5 days were transduced with either shHOBIT or shControl lentiviruses. The percentages of CD56+ NK cells within the total cells at day 35 were assessed by flow cytometry. Percentages of CD56+ cells within the transduced GFP+ and non-transduced GFP- fractions are shown. Results are calculated from four independent experiments using different donor cells and displayed as mean +/- SEM.

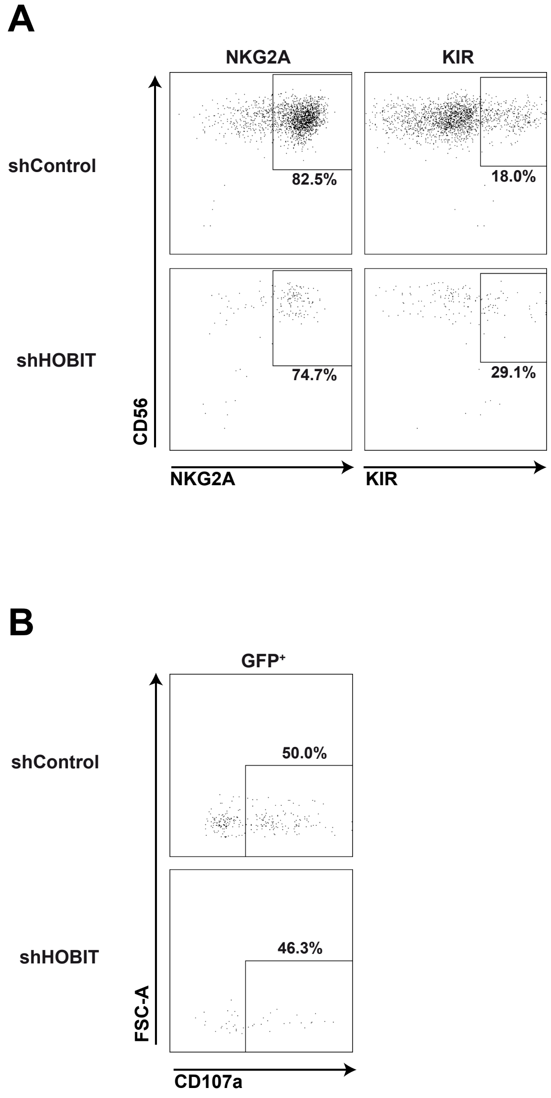


**Supplementary Figure 2. Exemplary dot plots for the effects of HOBIT shRNA on NKG2A and KIR receptor expression and degranulation capacity.**
Expanded cord blood stem cells were transduced and further differentiated until day 35. (A) Exemplary dot plots of flow cytometry analysis displaying NKG2A and KIR receptor expression. (B) Exemplary dot plots displaying CD107a surface levels. Cells were cocultured with K562 target cells in the presence of CD107a antibodies. Then the cells were fixed, stained with CD56 antibodies and subjected to flow cytometry.

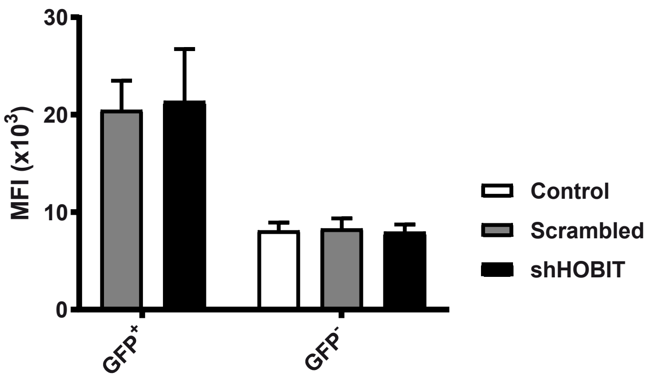


**Supplementary Figure 3. Effects of HOBIT shRNA on intracellular IFN- γ levels.** Expanded and transduced cord blood stem cells were differentiated until day 35. Cells were then cocultured with K562 target cells, permeabilized and stained with IFN-γ antibodies. The mean fluorescence intensity (MFI) of intracellular IFN-γ staining was assessed by flow cytometry as a measure for IFN-γ production per cell within the transduced GFP+ and non-transduced GFP- fractions. Results are derived from four independent experiments using different donor cells and are shown as mean +/- SEM.
